# Clustering analysis of risk factors for non-suicidal self-injury behaviors in adolescents: a cross-sectional study of western China

**DOI:** 10.3389/fpsyt.2025.1436868

**Published:** 2025-04-22

**Authors:** Xin Hou, Yuhang Wu, Jiayu Zhao, Jing Luo, Jinglong He, Qian Kang, Xuehua Li, Ling Yu, Lei Tang, Na Yong, Jiaming Luo

**Affiliations:** ^1^ Department of Psychiatry, The School of Psychiatry, North Sichuan Medical College, Nanchong, Sichuan, China; ^2^ Department of Psychiatry, The North Sichuan Medical College, Nanchong, Sichuan, China; ^3^ Department of Neurology, The Affiliated Hospital of North Sichuan Medical College, Nanchong, Sichuan, China; ^4^ Department of Clinical Medicine, The North Sichuan Medical College, Nanchong, Sichuan, China

**Keywords:** adolescents, non-suicidal self-injury (NSSI) behavior, risk factors, clustering analysis, western China

## Abstract

**Background and objectives:**

The analysis of clustering characteristics of disease risk factors makes for the formulation of corresponding prevention and control policies, but the risk factors for non-suicidal self-injury (NSSI) behaviors in adolescents is not covered, so this study is intended to explore the clustering characteristics of risk factors for NSSI behaviors in adolescents in the multi-center primary and secondary schools in western China.

**Methods:**

Utilizing random cluster sampling method, a total of 13,784 primary and secondary school students who met the research standards were randomly selected as the survey subjects from January 2020 to January 2021, and the clustering situation of the seven risk factors (depression, anxiety, stress, low social support, tolerance, avoidance, and emotional venting) among the students was analyzed. The characteristics of the respondents with a high clustering degree of risk factors for NSSI behaviors were also identified with the hierarchical logistic regression analysis.

**Results:**

4.2% of the adolescents in western China were detected with NSSI behaviors in the past year; the risk factors were grouped into 4 clusters, ranging from level 0 to level 3, with each level including 7692 (55.8%), 3847 (27.9%), 1303 (9.5%) and 941 (6.8%) of the survey subjects, respectively. The results of the Cochran-Armitage trend test analysis showed that there existed a linear trend between the clustering degree of risk factors and the detection rate of NSSI behaviors (P<0.000); according to the hierarchical Logistic regression analysis, the clustering degree of risk factors for NSSI behaviors was higher in the adolescents whose parents divorced and remarried 1.21(0.016~0.373) and whose fathers received only primary school education or below 1.23 (0.005~0.404). By contrast, the degree was lower in the adolescents who are male 0.93(-0.132~-0.003) and had never attended boarding school 0.83(-0.286~-0.096), whose parents were not divorced 0.80 ( -0.367~-0.072 ), and whose fathers were farmers 0.87(-0.271~0.006).

**Conclusion:**

The risk factors for NSSI behaviors in adolescents are in clusters. As the risk factors continue to cluster, NSSI behaviors can be detected more easily in adolescents. With respect to the endeavors to prevent and control NSSI behaviors in adolescents, more attention should be focused on the mental health of the adolescents who are female and attend boarding schools, whose parents have broken marriages, and whose fathers have low literacy.

## 1 Introduction

Non-suicidal self-injury (NSSI) comprises deliberate and conscious self-injurious acts without the intention to die. These acts can cause immediate physical damage to the body tissue, and include behaviors such as cutting, scratching, biting, burning and hitting oneself (1). In most cases, the onset of NSSI behaviors and adolescence march in lockstep, and its prevalence rates increase gradually after 14 years old (2, 3) and decline slowly in young adulthood. A meta-analysis showed that the lifetime prevalence rate of NSSI among adolescents who are 10 to 17 years old was 17.2% (4) and more than 50% of the adolescents continued to harm themselves till their adulthood (5). In recent years, the detection rate of NSSI in China has also shown an upward trend, and some studies have found that the rate in Chinese middle school students is 22.37% (6). Apart from being one of the most powerful tools to predict suicide tendency, NSSI attributes mainly to the death of adolescents (7–9), which makes it a globally serious public health issue. Considering the serious harm NSSI inflicts on adolescents and its high prevalence and protracted course, it is of great clinical significance to understand the risk factors of NSSI before the first onset, so that it can be better prevented and controlled.

Previous NSSI models suggested that NSSI occurred when individuals felt angry, self-blamed, depressed, frustrated and other negative emotions. In the case of emotional management barriers such as inability to express emotions, difficulty in emotion regulation, high emotion intensity, and lack of emotion control strategies, they tend to take self-injurious behavior to avoid or alleviate unpleasant and disgusting emotional experiences when they have no good coping style to regulate negative emotions[10, 11). Besides, a large number of studies also support that emotional management ability and negative coping style are important influencing factors of NSSI (11–13). At the same time, many scholars have also pointed out that negative emotions, such as anxiety and depression, and corresponding emotion–focused coping strategies like avoidance, tolerance, and venting of emotions, are risk factors for NSSI (14–16), and the negative emotions and coping strategies are believed to interact with the predictors of NSSI. Although all the above risk factors indicate an increased risk of NSSI behaviors, previous studies have shown that risk factors are often clustered, and with the increase of associated risk factors in number, the incidence of NSSI increases (17).

The clustering of risk factors means two or more than two risk factors turn up in an individual at the same time. The Clustering of risk factors means that a disease is not caused by a single or several causes, but the result of a combination of multiple risk factors, and these risk factors tend to Clustering. Moreover, there is an interaction between these risk factors. Independent risk factors have the effect of risk superposition and positive interaction. The interaction of multiple risk factors is much higher than that of single risk factors. Contemporary studies on the clustering analysis of risk factors displayed that a certain risk factor is often accompanied by another, which indicated that the expected results may be unavailable if research is done on a single risk factor, so the studies concluded that better cost benefits could be obtained should research be expanded to multiple risk factors study instead of focusing on a single risk factor (18, 19). If timely interference can be made in some modifiable risk factors in a targeted manner, it is of great significance to prevent the onset of NSSI in adolescents and improve the prognosis. Currently, there is no research on the clustering situation of risk factors for NSSI at home and abroad. The west of China, known for its vast agrarian lands, backward industry and the weak economy, has a large number of left–behind children (20), whose mental health is faced with severe challenges. Therefore, focused on students from multi–center primary and secondary schools in western China, this study, with the aim to analyze the distribution and clustering situation of risk factors for NSSI, aspires to provide guidance on how to prevent and control NSSI in adolescents.

The number of left–behind children in western China is large (20). This region is a large agricultural area, an old industrial area, and an economically poor area. Studies have shown that children in economically underdeveloped areas or whose parents go out for a long time will show higher levels of stress, depression and anxiety, and are accustomed to using negative coping strategies (21, 22). Therefore, the mental health of adolescents in western China should be focused on.

Based on the above information, we put forward the following research hypotheses: 1) the risk factors for NSSI behaviors in adolescents are in clusters and as the risk factors continue to cluster, NSSI behaviors are to be detected more easily in adolescents (2), relevant factors possibly affecting the clustering degree of risk factors for NSSI behaviors in adolescents are screened out. Incorporating related individual and sociological factors for NSSI in a fully systemic manner, this study is to explore the risk factors for NSSI behaviors and their clustering situation so as to provide research approaches and scientific support for early identification and prevention of self–injury behaviors in adolescents.

## 2 Respondent and methods

### 2.1 Respondents

Studies have shown that NSSI behavior mainly occurs in 10–17 years old, and the incidence rate gradually decreases after 18 years old (4). Therefore, this study takes adolescents aged 10–17 as the research object.

This study is a multicenter cross–sectional study conducted in western China from January 2020 to January 2021. The study takes cities, districts, and schools as sampling units, and uses a random cluster sampling method to involve 10–17 years old students from 50 schools in Sichuan Province and surrounding provinces and cities. Inclusion criteria: 1. Students aged 10 to 17 years old, 2. Han nationality,

Exclusion criteria: 1. Patients who are unable to accept the survey due to mental illness such as schizophrenia and intellectual disability, 2. Combined with epilepsy, severe and severe asthma, heart disease and other serious physical diseases, 3. Parents and students refuse the study.

The study has been approved by the ethics committee of north Sichuan medical college. All Parents signed the informed consent.

### 2.2 Research methods

Under the organization and coordination of Nanchong Municipal Bureau of Education and Sports, the on–site survey was jointly carried out by the Psychological Crisis Intervention Working Group of Mental Health Center in the Affiliated Hospital of North Sichuan Medical College, together with the work team. Before the survey, a pilot survey was conducted in a school other than the one selected for the survey.

Before the formal investigation, principals of all selected schools were organized to attend a symposium where the significance, workflow, precautions and quality requirements of this survey were introduced.

The survey process is as follows: invite Psychiatry and Epidemiology relevant experts inside and outside the hospital to participate in the questionnaire design, establish the initial questionnaire, and generate the final questionnaire after three rounds of discussion. Set up a special investigation team to conduct questionnaire training for the investigators, inform them of the purpose, significance and precautions of the investigation, unify the investigation procedures and methods, and undertake the on–site investigation only after passing the training. During the investigation, we communicated with the principals, teachers and students of the schools under investigation in advance to obtain support and cooperation. During the investigation, the class was the unit, and with the assistance of the head teacher, the questionnaire was distributed by the investigators. Before the subjects were filled in, we used unified guidelines to explain the purpose and significance of the investigation, filling methods and precautions, and told them that the investigation was anonymous, so as to ensure that the research data was true and effective. All the questionnaires were completed by the students themselves on the spot within the specified time and checked by the investigators before they were taken back. All respondents and guardians were investigated with written informed consent.

This study uses code instead of identity information such as name to ensure data confidentiality and participant anonymity. Four investigators timely reviewed the data and entered it into Excl, deleted the questionnaires with missing key information, missing questionnaires or the same answers, and checked and handled the missing and abnormal values to ensure the accuracy of the data. A total of 21433 questionnaires were collected in this survey, including 19258 valid ones and 19258 invalid ones, with an effective rate of 89.9%. After the inclusion and exclusion criteria, a total of 13784 questionnaires were included in the further study.

### 2.3 Research instrument

The survey was conducted with a self–made questionnaire on general information, depression–anxiety–stress scale (DASS–21) and coping style scale for middle school students (CSSMSS).

- The general demographic data questionnaire was used, including gender, grade, age, whether the only child, parents ‘ cultural situation, parents ‘ marital status, parents ‘ occupation, whether left–behind children and so on. Left–behind children were defined as children are left in the place of household registration for more than half for both parents or one of them go out to work.
- The depression–anxiety–stress scale (DASS–21), made up of 3 sub–scales, examines an individual’s experience of depression, anxiety and negative emotions resulting from stress, respectively. Each sub–scale contains 7 items which are graded on a 4–level scale, with “not suitable” as 0 points, “sometimes suitable” as 1 point, “often suitable” as 2 points, “always suitable” as 3 points. DASS–21 The scale consists of three subscales, which are depression (item 3,5,10,13,16,17,21), anxiety (item 2,4,7,9,15,19,20), and stress (item 1,6,8,11,12,14,18). Depression score > 9 points indicates depression, anxiety score > 7 points indicates anxiety, stress score > 14 points indicates stress. The coefficient and retest reliability of Cronbach’s α of the Chinese version of the scale are 0.912 and 0.751, respectively, with high reliability and validity (23).
- The coping Style Scale for Middle School Students (CSSMSS) consists of two sub–scales. One is the “problem–focused coping” sub–scale which includes the three factors of “problem–solving”, “seeking social support” and “positive and rationalized explanation”. The other is the “emotion–focused coping” sub–scale which includes the four factors of “tolerance”, “avoidance”, “venting of emotions” and “denial of fantasy”. Each factor is comprised of several items which are scored on a 4–level scale, with “not use” as 1 point, “use occasionally” as 2 points, “use sometimes” as 3 points and “use frequently” as 4 points. The sum of item scores makes the scores of factors, and the addition of factor scores belonging to the same sub–scale is the score of the sub–scale. Generally, the total score of the CSSMSS is not measured. The reliability and validity of the scale are good. Cronbach α coefficient of the total scale is 0.92, and the coefficient of retest reliability is 0.89 (24).
- Ottawa Self–Injury Inventory can conduct a comprehensive assessment of NSSI behavior in adolescents, including the site of NSSI, the reasons for selecting the site, the purpose of NSSI behavior, the reasons for the current existence of NSSI behavior and the measurement of potential addiction characteristics. The internal consistency coefficient was 0.67–0.87. In the study, the scale was used to assess the NSSI behavior of adolescents (25).

NSSSI was defined as 10 ways of self–injury without suicidal thoughts in the past 1 year, such as hitting the wall, biting yourself, picking or scratching yourself, pulling your hair hard, stabbing yourself with a knife or sharp weapon, and tying yourself with a rope. NSSI behavior is defined as NSSI that occurs once or more In the past 1 year.

### 2.4 Indices and their definitions

Previous studies have shown that negative emotions and negative coping styles are important influencing factors of NSSI. The NSSI model indicates that psychological or self–regulation factors are considered as the proximal and core factors of self–injury (11). Therefore, the following seven risk factors are set according to previous literature (14–16) and they are depression (defined as depression dimension score > 9 points in DASS–21), anxiety (defined as anxiety dimension score > 7 points in DASS–21), stress (defined as stress dimension score > 14 points in DASS–21), low social support (defined as social support dimension score ≤ 12 points in CSSMSS, based on the national norm of 16.48 ± 4.04 points), tolerance (defined as tolerance dimension score ≥ 12 points in CSSMSS, based on the national norm of 8.94 ± 2.42 points), avoidance (defined as avoidance dimension score ≥ 10 points in CSSMSS, based on the national norm of 7.23 ± 2.36 points), emotional venting (defined as emotional venting dimension score ≥11 points in CSSMSS, based on the national norm of 7.70 ± 2.50 points) (26).

The clustering of risk factors: according to the number of risk factors, the survey subjects were divided into 4 clusters marked from level 0 to level 3, of which level 0 meant none of the above risk factors was manifested in the subject, and level 3 meant three or more than three risk factors manifested themselves in the subject at the same time (27).

### 2.5 Statistical analysis

The database was built with Epidata3.1 and data analysis was conducted with SPSS23.0. The measurement data were described with mean ± standard deviation, the comparison between groups was carried out with student’s test, and Non–parametric test (Kruskal– Wallis rank sum test) was used to compare the distribution of combined risk factors in different populations. Cochran–Armitage trend (CAT) test was used to analyze the trend. During multivariate analysis, by virtue of logistic regression, the dependent variable was whether or not NSSI behaviors occurred, stepwise regression analysis was used to select variables, and the results were expressed as OR (95% CI).

## 3 Research results

### 3.1 General information

A total of 13,784 primary and secondary school students were under investigation. Among them, NSSI behaviors once occurred to 584 students, with the detection rate being 4.2%. Among all the survey subjects, there were 7128 male students (51.7%), and 6655 female students (48.3%), with 5653 students (41.0%) coming from rural areas, and 36.4% students being “left–behind” children. There were 13% of students whose parents divorced, and 7.3% of students who don’t live with their parents for a long time. With regard to the educational background of students’ fathers, 2,344 (17.0%) of them attended primary school or below. Regarding the educational background of students’ mothers, 3785 (27.5%) of them attended primary school or below. See **Table 1** for details.

**Table 1:** General demographic data.

Factors		Number	Percentage (%)
Sex	Male	7128	51.7
	Female	6655	48.3
Only Child	Yes	1904	13.8
	No	11879	86.2
Residence	Countryside	5653	41.0
	City	8130	59.0
Boarding	Yes	2379	17.3
	No	11404	82.7
“Left–behind” Children	Yes	5023	36.4
	No	8760	63.6
Parental Marriage	Remarried	692	5.0
	Single parent	1093	7.9
	Parents	11998	87.0
With Whom does he/she live?	Parents	11998	87.0
	Father	346	2.5
	Mother	435	3.2
	Grandparent(s)	1004	7.3
Father’s Occupation	Employee in a(n) enterprise or public institution	2256	16.4
	Farmer	3248	23.6
	Civil servant	116	0.8
	Individual businessman	1285	9.3
	Others	6878	49.9
Father’s Qualification	Primary school or below	2344	17.0
	Secondary school	8412	61.0
	High school or specialized secondary school	2288	16.6
	Junior college or above	739	5.4
Mother’s Occupation	Employee in a(n) enterprise or public institution	2049	14.9
	Farmer	3607	26.2
	Civil servant	47	0.3
	Individual businessperson	1066	7.7
	Others	7014	50.9
Mother’s Qualification	Primary school or below	3785	27.5
	Secondary school	7794	56.5
	High school or specialized secondary school	1737	12.6
	Junior college or above	467	3.4

### 3.2 Analysis of distribution of risk factors for NSSI behaviors in adolescents

Adolescents with NSSI behaviors obtained significantly higher scores in anxiety, depression, and stress than those without NSSI behaviors, which demonstrates a statistical difference (P<0.001). Adolescents with NSSI behaviors obtained significantly higher scores in the emotion–focused coping style which includes avoidance, tolerance, and venting of emotions than those without NSSI behaviors (P<0.001), but in terms of the scores in social support, the condition was rather opposite. The difference was statistically significant (P<0.001). The differences showed that depression, anxiety, stress, low social support and coping strategies like tolerance, avoidance, and emotional venting are all risk factors for NSSI behaviors in adolescents in western China. See **Table 2** for details.

**Table 2:** Analysis of distribution of risk factors for NSSI behaviors in adolescents.

Risk factors	Without NSSI (n=13199)	With NSSI (n=584)	T	P
Depression	2.15 ± 2.82	7.11 ± 5.12	–39.706	0.000
Anxiety	1.95 ± 2.67	6.26 ± 4.56	–36.696	0.000
Stress	2.77 ± 3.20	7.32 ± 4.85	–32.729	0.000
Social Support	17.68 ± 4.64	16.08 ± 4.50	8.394	0.000
Tolerance	8.71 ± 2.69	10.10 ± 2.64	–12.390	0.000
Avoidance	7.25 ± 2.59	8.33 ± 2.60	–9.803	0.000
Emotional Venting	7.18 ± 2.69	8.85 ± 3.09	–14.564	0.000

### 3.3 Trend analysis of detection rate of NSSI behaviors in adolescents as clustering degree of risk factors changes

Among all the survey subjects, there were 7692 (55.8%), 3847 (27.9%), 1303 (9.5%) and 941 (6.8%) students respectively located in the four clustering levels of risk factors from level 0 to level 3. Cochran–Armitage trend test analysis showed that a linear trend existed between the clustering degree of risk factors and the detection rate of NSSI behaviors (P<0.000), which meant that as clustering degree of the seven risk factors continuously increased, the detection rate of NSSI in adolescents accordingly rose significantly, as shown in **Table 3**.

**Table 3:** Trend analysis of detection rate of NSSI behaviors in adolescents as clustering degree of risk factors changes.

Risk factor combination (s)	0	1	2	≥3	EXP(B)	P
Without NSSI	7552 (57.2)	3694 (28.0)	1196 (9.1)	757 (5.7)	0.044	<0.000
With NSSI	140 (24.0)	153 (26.2)	107 (18.3)	184 (31.5)	2.346	<0.000
Aggregate	7692	3847	1303	941		

### 3.4 Univariate analysis of clustering degree of risk factors for NSSI behaviors in adolescents

The results of univariate analysis showed that such factors as sex, family residence, boarding or not, “left–behind” children or not, parents’ marital status, parents’ occupations and educational levels all had an impact on the clustering degree of risk factors, and the differences were statistically significant (P<0.05). See **Table 4**.

**Table 4:** Univariate analysis of clustering degree of risk factors for NSSI behaviors in adolescents.

Factors	Risk factor combination (s)				X2	P
	0	1	2	≥3		
Sex						
Male	4021 (56.4)	1997 (28.0)	668 (9.4)	442 (6.2)	4.191	0.041
Female	3714 (55.2)	1850 (27.8)	635 (9.5)	499 (7.5)		
Only Child						
Yes	1071 (13.9)	494 (12.8)	193 (14.8)	146 (15.5)	0.093	0.760
No	6621 (86.1)	3353 (87.2)	1110 (85.2)	795 (84.5)		
Residence						
Countryside	3043 (39.6)	1613 (41.9)	581 (44.6)	416 (44.2)	18.105	0.000
City	4649 (60.4)	2234 (58.1)	722 (55.4)	525 (55.8)		
Boarding						
Yes	1192 (15.5)	694 (18.0)	265 (20.3)	228 (24.2)	50.808	0.000
No	6500 (84.5	3153 (82.0)	1038 (79.7)	713 (75.8)		
“Left–behind” Children						
Yes	2720 (35.4)	1405 (36.5)	511 (39.2)	387 (41.1)	13.349	0.000
No	4972 (64.6)	2442 (63.5)	792 (60.8)	554 (58.9)		
Parental Marriage						
Remarried	6819 (88.7)	3316 (86.2)	1095 (84.0)	768 (81.6)	54.615	0.000
Single parent	326 (4.2)	197 (5.1)	85 (6.5)	84 (8.9)		
Parents	547 (7.1)	334 (8.7)	123 (9.4)	89 (9.5)		
With Whom does he/she live?						
Parents	6891 (88.7)	3316 (86.2)	1095 (84.0)	768 (81.6)	1.844	0.398
Father	164 (2.1)	102 (2.7)	49 (3.8)	31 (3.3)		
Mother	225 (2.9)	122 (3.2)	51 (3.9)	37 (3.9)		
Grandparent (s)	484 (6.3)	307 (8.0)	108 (8.3)	105 (11.2)		
Father’s Occupation						
Employee in a (n) enterprise or public institution	1238 (16.1)	615 (16.0)	213 (16.3)	190 (20.2)	13.477	0.009
Farmer	1883 (24.5)	894 (23.2)	296 (22.7)	175 (18.6)		
Civil servant	65 (0.8)	31 (0.8)	9 (0.7)	11 (1.2)		
Individual businessman	731 (9.5)	339 (8.8)	131 (10.1)	84 (8.9)		
Others	3775 (49.1)	1968 (51.2)	654 (50.2)	484 (51.5)		
Father’s Qualification						
Primary school or below	1187 (15.4)	686 (17.8)	254 (19.5)	217 (23.1)	44.814	0.000
Secondary school	4733 (61.5)	2354 (61.2)	782 (60.0)	543 (57.7)		
High school or specialized secondary school	1347 (17.5)	604 (15.7)	206 (15.8)	131 (13.9)		
Junior college or above	425 (5.5)	203 (5.3)	61 (4.7)	50 (5.3)		
Mother’s Occupation						
Employee in a (n) enterprise or public institution	1103 (14.3)	572 (14.9)	200 (15.3)	174 (18.5)	13.416	0.009
Farmer	2070 (26.9)	1006 (26.2)	334 (25.6)	197 (20.9)		
Civil servant	23 (0.3)	13 (0.3)	5 (0.4)	6 (0.6)		
Individual businessperson	600 (7.8)	291 (7.6)	108 (8.3)	67 (7.1)		
Others	3896 (50.7)	1965 (51.1)	656 (50.3)	497 (52.8)		
Mother’s Qualification						
Primary school or below	2004 (26.1)	1082 (28.1)	408 (31.3)	291 (30.9)	24.907	0.000
Secondary school	4397 (57.2)	2180 (56.7)	708 (54.3)	509 (54.1)		
High school or specialized secondary school	1022 (13.3)	455 (11.8)	153 (11.7)	107 (11.4)		
Junior college or above	269 (3.5)	130 (3.4)	34 (2.6)	34 (3.6)		

### 3.5 Multivariate analysis results of clustering degree of risk factors

As shown in **Table 5**, with the hierarchical logistic regression, such factors as whether being female, residing in the countryside, attending boarding school and whether being the “left–behind” children were taken as references. Studies showed that the clustering degree of risk factors for NSSI behaviors was higher in the adolescents who are females, whose parents divorced and remarried and whose fathers were farmers, whereas that was relatively low in the adolescents who are males, whose parents enjoyed a fair marriage and whose fathers attended only primary school or below.

**Table 5:** Multivariate analysis results of clustering degree of risk factors for NSSI behaviors in adolescents.

Independent Variable		B value	OR value (95%)	P value
Sex		0.033	0.93 (–0.132~–0.003)	0.042
Residence in countryside or not		0.040	1.03 (–0.047~0.108)	0.442
Boarding		0.049	0.83 (–0.286~–0.096)	0.000
“Left–behind” Children		0.037	0.99 (–0.081~0.065)	0.828
Parental Marriage	Divorced & single parent			
	Parents	0.061	0.80 (–0.367~–0.072)	0.000
	Divorced & remarried	0.091	1.21 (0.016~0.373)	0.033
Father’s Occupation	Others			
	Employee in a (n) enterprise or public institution	0.061	1.02 (–0.104~.134)	0.809
	Farmer	0.068	0.87 (–0.271~–0.006)	0.041
	Civil servant	0.192	1.07 (–0.311~0.441)	0.734
	Individual businessman	0.076	0.99 (–0.161~0.138)	0.880
Father’s Qualification	Junior college or above			
	Primary school or below	0.102	1.23 (0.005~0.404)	0.045
	Secondary school	0.093	1.01 (–0.173~0.192)	0.918
	High school or specialized secondary school	0.095	0.94 (–0.248~0.123)	0.510
Mother’s Occupation	Others			
	Employee in a (n) enterprise or public institution	0.063	1.11 (–0.015~0.230)	0.086
	Farmer	0.066	0.99 (–0.138~0.121)	0.893
	Civil servant	0.283	1.48 (–0.164~0.947)	0.167
	Individual businessperson	0.082	1.06 (–0.103~0.218)	0.481
Mother’s Qualification	Junior college or above			
	Primary school or below	0.117	1.22 (–0.034~0.425)	0.096
	Secondary school	0.113	1.12 (–0.105~0.336)	0.304
	High school or specialized secondary school	0.115	1.04 (–0.189~0.261)	0.754

## 4 Discussion

This study is the first to conduct a large–scale clustering analysis of the risk factors for NSSI behaviors in adolescents from several schools in western China, which can make for a deep and detailed exploration of the status quo and feature distribution of NSSI problems in adolescents. This study reveals that the risk factors for NSSI behaviors in adolescents are in clusters and as the clustering degree increases, the risk of NSSI behaviors in adolescents rises accordingly. Moreover, such factors as sex, parents ‘ marital status, father’s educational background and occupation are all related to the clustering degree of risk factors for NSSI behaviors in adolescents.

In this study, the detection rate of NSSI in the last year was 4.2%. A recent study of underdeveloped areas in western China suggested that the detection rate of NSSI in children and adolescents was 47% (28). A survey of 2200 middle school students in Shenzhen showed that the detection rate of NSSI behavior was 10.9% (29), while another meta–analysis showed that the overall detection rate of NSSI among middle school students in Chinese Mainland was 27.4% (30), which may be attributed to the discrepancies in the age of the targeted subjects, questionnaires, definitions of indices, social factors (31), as well as early implementation of mental health screening in primary and secondary schools in the west of China.

Among all the respondents in this survey, the proportions with 1, 2 and ≥ 3 risk factors are 27.9%, 9.5% and 6.8%, respectively. Nearly half of the adolescents have NSSI risk factors, and 16.3% of the population have NSSI risk factors, indicating that there is a crisis in the mental health of adolescents in western China. A recent survey on the risk factors for adult mental health showed that 19.34%, 24.37%, 18.98% and 27.66% of the respondents were plagued by one, two, three, four or more than four risk factor(s), respectively (32). Another study on adolescents with NSSI behaviors displayed that 4. 15%, 7.82%, 18. 15% and 31. 10% of the respondents were disturbed by one, two, three, four or more than four risk factor(s), respectively (33). Previous studies have shown that the clustering degree of risk factors varies among different populations in different regions, and generally the clustering degree in this study is lower than that in previous studies. Although a huge gap exists between the

results of different studies due to diverse risk factors and demographic differences of survey subjects, all the results suggest an obvious clustering tendency of risk factors.

What’s more, the study suggests that high clustering degree of risk factors represents high detection rate of NSSI behaviors in adolescents. Among adolescents with NSSI behaviors, 49.8% of them are beset by two or more than two risk factors.

Some studies also found that the risk factors, such as smoking, drinking, being bullied at school, heavy learning pressure, among others, for NSSI behaviors in rural high school students cluster and with the increase of clustering degree of risk factors, the risk of NSSI behaviors in them also rises. It’s found that when risk factors reach three or over in number, the risk of NSSI behaviors increases by 12.6 times (17). Scholars also indicated that more risk factors of mental health meant a higher risk of psychological distress (34–36). The collaboration or interaction between multiple risk factors that coexist tends to increase significantly the risk of NSSI behaviors [19.20], so the endeavors to screen people with multiple risk factors should be strengthened insomuch that key prevention and control efforts can be carried out smoothly.

The results of multivariate analysis show that risk factors for NSSI behaviors in females tend to cluster more significantly than in males. Most studies showed that the incidence of NSSI behaviors in the female in the past or just within the past year was higher than that in the male (37, 38) which may be ascribed to the fact that females in puberty are more easily affected by the external environment or negative emotions (39), and they are inclined to adopt negative attitudes, like avoidance, towards the external stress. Girls in remote cities in western China are more vulnerable to the influence of traditional Chinese ideas and are introverted. Women enter puberty earlier than boys and face more psychological problems and problems. Girls often adopt emotion focusing strategies and boys use cognitive reappraisal strategies. As a

result, girls ‘ regulation effect is not as good as boys’, and they are prone to be anxious, depressed and have other negative emotions, causing more psychological problems (40, 41).

This study also points out that the risk factors for NSSI behaviors cluster even more in adolescents who attended boarding school, whose parents divorced and remarried, and whose fathers attended only primary school or below. Some studies showed that due to a constant lack of familial comfort and care, many boarding students are more liable to mental health problems since they often suffer from such negative feelings as tension and anxiety, a sense of insecurity. With poor interpersonal skills, they tend to feel insecure in society and thus give no trust in it, thereby receiving less social support when they are in need (42). The lower educational qualification of the fathers indicates their infrequent and negative participation in cultivating the children, which is likely to invite an increased risk of depression, anxiety, hostility towards society, and discomfort in society in children in the future (43). Meanwhile, the mental health status of middle school students and their family intimacy are positively correlated. The breakdown of family relationships caused by different reasons contributes significantly to the children’s psychological problems. Studies revealed that when parents are mutually hostile and aggressive in a family, the child tends to be mentally stressed and confused, thus their perception of external

support reduced (44). Adolescents with a broken family are more prone to emotional problems and coping style problems, so the incidence of NSSI behaviors in them is also higher (45).

Conversely, the clustering degree of risk factors for NSSI behaviors is relatively low in adolescents who are male, whose parents have a sound marriage and whose fathers are farmers. The relatively low clustering degree of risk factors for NSSI behaviors in adolescents whose fathers are farmers may result from the low academic requirements the families set for the children who, therefore, face less stress and negative responses related to academic learning. Furthermore, some scholars pointed out that a father’s occupation as a farmer represents the weak economic strength and low social status of the family in China, which directly affect the investment of household income and social capital, as well as parenting effectiveness, so that the children are faced with higher risks of psychological and behavioral disorders (46–48), more liable to psychological problems and behaviors harmful to themselves or others (49). Possible reasons for this phenomenon are the rise in social status and income of the farmers with the reform and development of China’s economy and society.

This study is the first to analyze the distribution and aggregation of NSSI risk factors among primary and secondary school students and screen out the relevant factors that affect the aggregation of NSSI risk factors. The advantage lies in the large range and large sample size, which ensures sufficient statistical ability. At the same time, standardized questionnaires and well–trained investigators are used to collect data on campus, and good quality control ensures the validity of data. This study discovers that endeavors to screen people baffled by multiple risk factors should be strengthened and key efforts to prevent and control the risk factors should be emphasized, which is rather significant for the policy–makers who intend to work out the mental health problems of adolescents. Accordingly, in the midst of efforts to prevent and control NSSI behaviors in adolescents, more attention should be focused on the adolescents who are female, boarding students, whose parents have broken marriages and whose fathers’ educational qualifications are at disadvantage. Actively carry out health education and psychological counseling activities for key personnel, strengthen the attention of parents, establish mental health files, and regularly check psychological status to reduce the occurrence of NSSI behavior. We have added the corresponding information in the newly submitted manuscript.

This study has shortcomings in that data were collected in only a portion of western China and did not cover all cities and schools, which could be expanded in future studies. Also, reporting and recall bias may be unavoidable because questionnaires were administered in the form of self–assessment scales and retrospective reports. In addition, this study was a cross–sectional study, and it was not possible to determine the possible changes between risk factor clustering and NSSI over time, and further research is needed. In the future, longitudinal study design can be used to conduct long–term follow–up on adolescent mental health, so as to better screen out high–risk groups who implement NSSI behavior for intervention. We have added the corresponding information in the newly submitted manuscript.

## 5 Conclusions

To sum up, the study results suggest that the risk factors for NSSI behaviors cluster clearly in adolescents in western China, and as the clustering degree of risk factors increases, the risk of NSSI behaviors rises accordingly. The study also displays that in the midst of efforts to prevent and control NSSI behaviors in the future, key emphasis should be put on the adolescents who are female, boarding students, whose parents have broken marriages and whose fathers have poor literacy.

## Data Availability

The original contributions presented in the study are included in the article/supplementary material. Further inquiries can be directed to the corresponding author.
